# Clinical and Molecular-Based Approach in the Evaluation of Hepatocellular Carcinoma Recurrence after Radical Liver Resection

**DOI:** 10.3390/cancers13030518

**Published:** 2021-01-29

**Authors:** Salvatore Gruttadauria, Floriana Barbera, Pier Giulio Conaldi, Duilio Pagano, Rosa Liotta, Enrico Gringeri, Roberto Miraglia, Gaetano Burgio, Marco Barbara, Giada Pietrosi, Calogero Cammà, Fabrizio Di Francesco

**Affiliations:** 1 Department for the Treatment and Study of Abdominal Diseases and Abdominal Transplantation, Istituto di Ricovero e Cura a Carattere Scientifico—Istituto Mediterraneo per i Trapianti e Terapie ad Alta Specializzazione (IRCCS-ISMETT), University of Pittsburgh Medical Center (UPMC), 90127 Palermo, Italy; dpagano@ismett.edu (D.P.); gpietrosi@ismett.edu (G.P.); fdifrancesco@ismett.edu (F.D.F.); 2 Department of Surgery and Medical and Surgical Specialties, University of Catania, 95124 Catania, Italy; 3 Research Department, Istituto di Ricovero e Cura a Carattere Scientifico—Istituto Mediterraneo per i Trapianti e Terapie ad Alta Specializzazione (IRCCS-ISMETT), 90127 Palermo, Italy; fbarbera@ismett.edu (F.B.); pgconaldi@ismett.edu (P.G.C.); mbarbara@ismett.edu (M.B.); 4 Pathology Unit, Department of Diagnostic and Therapeutic Services, Istituto di Ricovero e Cura a Carattere Scientifico—Istituto Mediterraneo per i Trapianti e Terapie ad Alta Specializzazione (IRCCS-ISMETT), 90127 Palermo, Italy; rliotta@ismett.edu; 5 Hepatobiliary Surgery and Liver Transplantation Unit, Department of Surgery, Oncology and Gastroenterology, University of Padova, 35122 Padova, Italy; enrico.gringeri@unipd.it; 6 Radiology Unit, Department of Diagnostic and Therapeutic Services, Istituto di Ricovero e Cura a Carattere Scientifico—Istituto Mediterraneo per i Trapianti e Terapie ad Alta Specializzazione (IRCCS-ISMETT), 90127 Palermo, Italy; rmiraglia@ismett.edu; 7 Department of Anesthesia and Intensive Care, Istituto di Ricovero e Cura a Carattere Scientifico—Istituto Mediterraneo per i Trapianti e Terapie ad Alta Specializzazione (IRCCS-ISMETT), 90127 Palermo, Italy; gburgio@ismett.edu; 8 Section of Gastroenterology & Hepatology, Department of Health Promotion, Mother and Child Care, Internal Medicine and Medical Specialties (PROMISE), University of Palermo, 90127 Palermo, Italy; calogero.camma@unipa.it

**Keywords:** HCC, liver resection, HCC recurrence, next-generation sequencing, loss of heterozygosity

## Abstract

**Simple Summary:**

Hepatocellular carcinoma (HCC) recurrence is still a major issue after liver resection. While several clinical factors were found to be associated with tumor recurrence, HCC pathogenesis is a complex process of accumulation of somatic genomic alterations, which has not been completely understood, especially with respect to tumor recurrence. The aim of this study is to complement potentially predictive clinical factors with next-generation sequencing genomic profiling and loss of heterozygosity analysis. We confirmed that serum bilirubin level, number of HCC nodules and size of the larger nodule are linked to a higher risk of tumor recurrence. Loss of heterozygosity in the *PTEN* loci was found to be associated with a lower risk of HCC recurrence.

**Abstract:**

Background: Hepatic resection remains the treatment of choice for patients with early-stage HCC with preserved liver function. Unfortunately, however, the majority of patients develop tumor recurrence. While several clinical factors were found to be associated with tumor recurrence, HCC pathogenesis is a complex process of accumulation of somatic genomic alterations, which leads to a huge molecular heterogeneity that has not been completely understood. The aim of this study is to complement potentially predictive clinical and pathological factors with next-generation sequencing genomic profiling and loss of heterozygosity analysis. Methods: 124 HCC patients, who underwent a primary hepatic resection from January 2016 to December 2019, were recruited for this study. Next-generation sequencing (NGS) analysis and allelic imbalance assessment in a case-control subgroup analysis were performed. A time-to-recurrence analysis was performed as well by means of Kaplan–Meier estimators. Results: Cumulative number of HCC recurrences were 26 (21%) and 32 (26%), respectively, one and two years after surgery. Kaplan–Meier estimates for the probability of recurrence amounted to 37% (95% C.I.: 24–47) and to 51% (95% C.I.: 35–62), after one and two years, respectively. Multivariable analysis identified as independent predictors of HCC recurrence: hepatitis C virus (HCV) infection (HR: 1.96, 95%C.I.: 0.91–4.24, *p* = 0.085), serum bilirubin levels (HR: 5.32, 95%C.I.: 2.07–13.69, *p* = 0.001), number of nodules (HR: 1.63, 95%C.I.: 1.12–2.38, *p* = 0.011) and size of the larger nodule (HR: 1.11, 95%C.I.: 1.03–1.18, *p* = 0.004). Time-to-recurrence analysis showed that loss of heterozygosity in the *PTEN* loci (involved in the PI3K/AKT/mTOR signaling pathway) was significantly associated with a lower risk of HCC recurrence (HR: 0.35, 95%C.I.: 0.13–0.93, *p* = 0.036). Conclusions: multiple alterations of cancer genes are associated with HCC progression. In particular, the evidence of a specific AI mutation presented in 20 patients seemed to have a protective effect on the risk of HCC recurrence.

## 1. Introduction

Hepatocellular carcinoma (HCC) is the fourth cause of cancer death worldwide and the most common primary liver cancer [1]. Along with thermal ablation and liver transplantation, hepatic resection (HR) is considered to be a first-line curative treatment [2,3,4,5,6,7]. Unfortunately, however, long-term prognosis remains inadequate because of the high rate of HCC recurrence (60 to 70%) in patients within 5 years after surgery [8]. Identifying risk factors of recurrence is important to improve long-term survival outcomes after HCC resection. Several clinical and pathological characteristics have been identified as predictors of tumor recurrence after HR, such as vascular tumor invasion, number and size of HCC nodules, alpha-fetoprotein level and tumor histological grading [8,9,10]. On the other side, HCC molecular pathogenesis is a complex process of accumulation of somatic genomic alterations, which lead to a huge molecular heterogeneity that has not been completely understood, and to our knowledge, no molecular classification has been successfully proposed yet for the prediction of tumor progression or recurrence [11].

Hepatocellular carcinoma shows a high degree of histological and molecular heterogeneity, including activation of PI3K/AKT and MAPK signaling pathways, TP53 mutations, overexpression of genes involved in the cell cycle and survival and chromosomal instability [12], as confirmed by whole-exome sequencing [13,14,15]. Moreover, genomic instabilities, such as small structure variations, microsatellite instability (MSI) and loss of heterozygosity (LOH), are characteristics of most tumor cells, including HCC [16,17,18,19,20].

The aim of this study was to evaluate a panel of specific microsatellites and mutations in HCC-specific genes in patients treated with partial hepatectomy for HCC, and to assess its potential role as predictor of HCC recurrence.

## 2. Materials and Methods

### 2.1. Study Objective and Endpoint

The main objective of the present study is to evaluate the strength of the association between the recurrence of HCC after HR and a set of pre-operative patient findings, including clinical and pathological characteristics, the presence of somatic variants in 26 cancer-related genes and the occurrence of LOH in a pre-specified panel of microsatellites. The main endpoint was time to HCC recurrence, defined as the number of days between HR and the first radiological evidence of tumor recurrence.

### 2.2. Study Populations and Design

All adult patients (aged 18 or more) with a histologically confirmed diagnosis of HCC who underwent a radical liver resection as first-line treatment from 1 January 2016 to 31 December 2019 at our institution were included for this study; patients were excluded in case of detection by the pathologist of microscopic tumor invasion of the resection margin (R1).

A subset of this population was subsequently selected for next-generation sequencing and loss of heterozygosity analyses following a case-control study design.

Specifically, among all patients with at least one year of follow-up at the moment of the extraction, 20 consecutive patients who had experienced HCC recurrence within one year after surgery were selected as cases; controls were then randomly extracted, without replacement, among those who underwent surgery in the same time window and had still not developed HCC recurrence.

### 2.3. Genomic DNA Extraction

DNA was purified from 10 unstained slides using QIAamp DNA FFPE Tissue kit (Qiagen, Germantown, MD, USA) and from whole blood using QIAamp DNA Mini kit (Qiagen, Germantown, MD, USA). The purity of the samples was determined by NanoDrop Spectrophotometer (Thermo Fisher Scientific, Waltham, MA, USA), the quality by Genomic DNA ScreenTape System (4200 TapeStation System, Agilent Technologies, Santa Clara, CA, USA) and concentration by Qubit dsDNA BR Assay kit (Thermo Fisher Scientific, Waltham, MA, USA)

### 2.4. Allelic Imbalance Analysis

Genomic DNA from each sample was amplified in a single PCR amplification assay using Type-it Multiplex PCR Master Mix (Qiagen, Germantown, MD, USA) and separated by capillary electrophoresis on 3500 Genetic Analyzer (Thermo Fisher Scientific, Waltham, MA, USA). A panel of 17 loci situated within or adjacent to specific genes of interest 1p (*L-myc*), 1p (*CMM*), 3p (*OGG1*), 5p (*MCC*), 9q (*PTCH*), 9p (*CDKN2A*/p16), 10q (*PTEN*), 17p (*TP53*) and 18q (*SMAD4*) was used to assess allelic imbalance associated with HCC recurrence (Table S1).

An allelic imbalance analysis investigates and compares peripheral blood cells with diseased tissue (neoplastic tissue) from the same patient. When a particular microsatellite marker in a sample manifested only a single peak, the microsatellite was designated as non-informative (homozygous, showing only 1 allele); while, when showing two peaks, it was defined as informative (heterozygous, showing 2 different alleles). Non-informative loci were excluded for frequency calculation of allelic imbalance. Signal intensity in tumor DNA was compared with those of the corresponding normal DNA using GeneMapper ID v4.1 software (Thermo Fisher Scientific, Waltham, MA, USA). Informative patients could be divided into 2 groups: group 1, including patients negative for LOH (tumor and normal DNA showed identical allelic patterns), and group 2, including patients with presence of LOH (reduction in the peak height of one of the two alleles of tumor DNA compared with normal DNA). The following equation is used to calculate the allele ratio (AR) between two allele peaks for each marker for each sample: AR = peak height of allele 1/peak height of allele 2. While the following equation is used to calculate the allelic imbalance (AI): AI = AR of healthy sample/AR of diseased sample. Alleles were assessed as being in balance (retention of heterozygosity) when AI values were within the range 0.66–1.50 (normal range). Values beyond this range (AI less than 0.66 or greater than 1.50) identified presence of AI.

### 2.5. Next-Generation Sequencing Analysis

The sequencing DNA libraries (genomic DNA input was 300 ng) were prepared using the TruSight Tumor 26 Kit (TST26, Illumina, San Diego, CA, USA), which allows for the detection of somatic variants in 174 amplicons covering 85 exonic regions in 26 cancer-related genes (Table S1). The sequencing 2 × 150 PE was performed with the MiSeq (Illumina, San Diego, CA, USA). Miseq Reporter software (Illumina, San Diego, CA, USA) was used for sequence alignment (human genome reference hg19 and TST26 manifest file), index and primer trimming and variant calls using a variant somatic caller. Quality check of raw reads was performed with FastQC tool. Genomic variants were annotated by means of the Illumina Variant Interpret tool. Analysis of the 26 tumor-related genes was performed after filtering genetic variants using the following criteria: PASS filter, frequency of alternative (Alt) allele (versus reference allele) ≥ 3%, variant call quality = 100 and read depth > 100. The TST26 panel can detect somatic alterations with VAF ≥ 3% [21] and less than 3% [22]. All variants were scrutinized to remove synonymous variants, leaving only variants affecting coding sequences (missense, InDel/frameshift, stop gained, stop lost, initiator codons, in-frame insertions, in-frame deletions, splice/intronic variants). The variants were evaluated according ClinVar Classification, in silico prediction tools (i.e., SIFT, and Polyphen), COSMIC database, Varsome tool. Each candidate variant in BAM files has been specifically evaluated using an integrative genomics viewer (IGV). Regions covered by Illumina TruSight Tumor 26 are shown in Table S2.

### 2.6. Statistical Analysis

Variables are summarized as frequency and percentage or as median and inter-quartile range (IQR), depending on their categorical or numerical scale. A preliminary analysis was performed on all variables to identify and check unusual values or outliers. Difference in the distribution of variables between two groups of patients were tested by means of the Pearson Chi-squared or Fisher exact test (as appropriate), if categorical, and by means of Student’s t-test or Mann–Whitney U test (as appropriate), if continuous. Kaplan–Meier estimates and curves were used to estimate the probability of HCC recurrence. Surgery date was used as time origin and time-to-recurrence was defined as the number of days between time origin and the first radiological evidence of tumor recurrence. Simple Cox proportional hazard models were used to estimate and test hazard ratios of HCC recurrence. The multivariable Cox model was obtained by means of a forward-stepwise procedure, using the best-AIC stopping rule; the resulting model was checked for the presence of correlation among the parameters using graphical inspection and testing interaction parameters. Hazard proportionality was verified using the Schoenfeld residual. All analyses and graphics were performed on the R statistical environment (version 4.0.2, R Core Team, Vienna, Austria).

## 3. Results

### 3.1. Preoperative Clinical Data

Over the study period, 124 patients with a histologically confirmed diagnosis of HCC underwent a radical (R0) partial hepatectomy at our center (Table 1). Ninety-six (77%) were male, the median age was 69 years (mean: 67, IQR: 62–73 years old) and the median body mass index (BMI) was 25.8 (mean: 26.4, IQR: 23–29). The most frequent etiology of liver disease was hepatitis C virus (HCV) infection (72 out of 124 patients, 58%), followed by non-alcoholic steatohepatitis (18 patients, 15%) and hepatitis B virus (HBV) infection (12 patients, 10%)

Most of the 72 patients with HCV etiology had been previously treated with Interferon + Ribavirin (37 out of 72 patients, 51%) and/or direct-acting antivirals (52 out of 72, 72%); 62 patients (86%) had achieved sustained virological response at the time of liver resection. All the patients with HBV infection were treated with Entecavir. Seventy-five percent of the patients had liver cirrhosis, and all patients but one were in class A of the Child–Pugh score, with a median model for end-stage liver disease (MELD) of 7 (mean: 7.8, IQR: 7–8).

### 3.2. Overall HCC Recurrence

**Figure 1:**
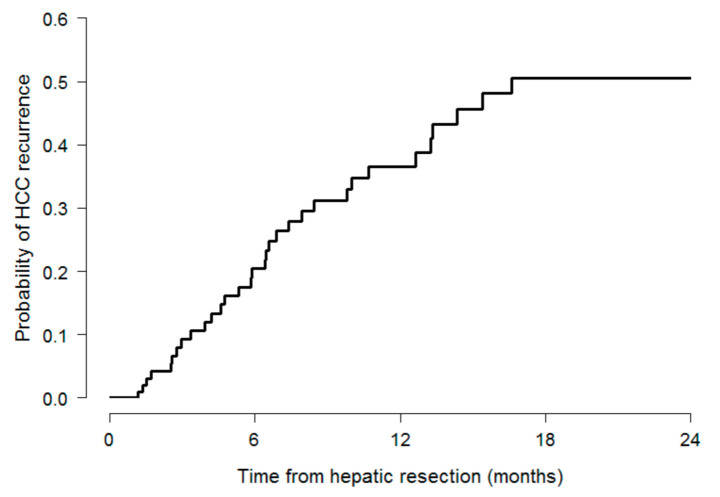
Kaplan–Meier estimates for the probability of HCC recurrence.

Cumulative numbers of HCC recurrences were 26 (21%) and 32 (26%), respectively, one and two years after surgery. Kaplan–Meier estimates for the probability of recurrence amounted to 37% (95% C.I.: 24–47) and to 51% (95% C.I.: 35–62), after one and two years, respectively (Table 1, Figure 1).

### 3.3. Associations between Preoperative Clinical Data and HCC Recurrence

Patients with a higher MELD score (HR: 1.43, 95%C.I.: 1.16–1.75, *p* < 0.001), higher serum bilirubin level (HR: 4.20, 95% C.I.: 1.74–10.14, *p* = 0.001), higher international normalized ratio level (HR: 177.6, 95% C.I.: 5.4–5872.0, *p* = 0.004), along with patients with more (HR: 1.81, 95% C.I.: 1.21–2.67, *p* = 0.003) an larger HCC nodules (HR: 1.09, 95% C.I.: 1.02–1.16, *p* = 0.011) have a statistically significant higher risk of developing HCC recurrence at the univariate time-to-recurrence analysis (Table 2).

The multivariable model maintained as independent predictors of HCC recurrence: HCV infection (HR: 1.96, 95% C.I.: 0.91–4.24, *p* = 0.085), serum bilirubin levels (HR: 5.32, 95% C.I.: 2.07–13.69, *p* = 0.001), number of nodules (HR: 1.63, 95% C.I.: 1.12–2.38, *p* = 0.011) and size of the larger nodule (HR: 1.11, 95% C.I.: 1.03–1.18, *p* = 0.004, Table 3).

### 3.4. Allelic Imbalance Analysis

**Figure A1:**
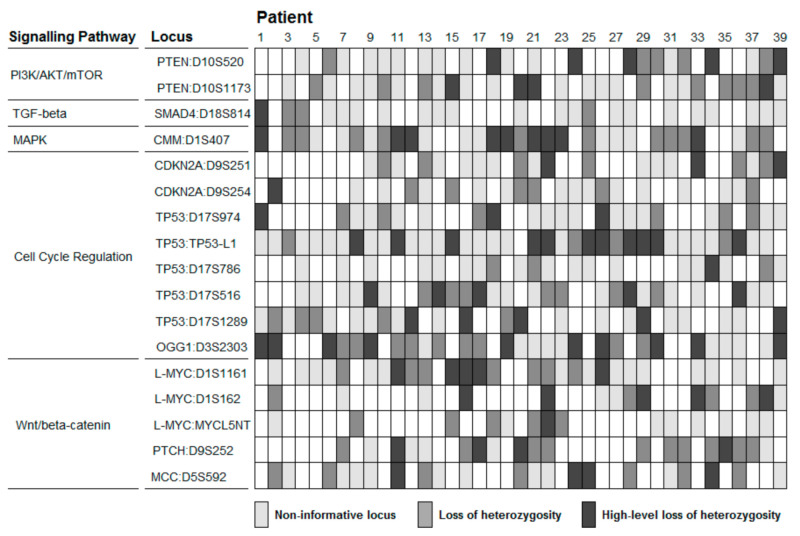
LoH analysis of 17 HCC related loci in 39 patients. Loss of heterozygosity is defined for values of allelic imbalance outside the range 0.66–1.50. High-level loss of heterozygosity is defined for values of allelic imbalance outside the range 0.50–2.00.

Loss of heterozygosity evaluation was performed in 39 patients, 19 of which from the group of cases and 17 from the group of controls (Table A2 and Figure A1). According to literature data about the association between the presence of AI in specific microsatellite loci and the risk of HCC recurrence, we analyzed 17 loci located within or adjacent to specific genes of interest: 1p (*L-myc*), 1p (*CMM*), 3p (*VHL* and *OGG1*), 5p (*MCC*), 5q (*APC*), 9q (*PTCH*), 9p (*CDKN2A*/p16), 10q (*PTEN*), 17p (*TP53*) and 18q (*SMAD4*). We compared the profiles of the loci in normal tissue (peripheral blood cells) and the corresponding neoplastic tissue. Time-to-recurrence analysis showed that LOH in the *PTEN* loci (involved in the PI3K/AKT/mTOR signaling pathway) was significantly associated with a lower risk of HCC recurrence (HR: 0.35, 95% C.I.: 0.13–0.93, *p* = 0.036, Table 4).

### 3.5. NGS Analysis Findings

The high-throughput sequencing was reliably obtained on 36 samples of hepatocellular carcinoma, 17 from the subgroup of cases (patient who developed HCC recurrence within 1 year after surgery) and 19 from the group of controls. We focused our attention only on somatic genetic alterations that occurred during neoplastic transformation and on those that modify the protein sequence, which could have an effect on protein coding and could be drivers in the acquisition of the tumor cells’ aggressive phenotype. We have identified somatic mutations with variant allele frequency (VAF) ranging from 3 to 49%. Only somatic variants passing the quality filter, with a minimum frequency of 3% and a read depth of at least 100 were considered. After interpretation workflow, variants annotated as synonymous, 3’UTR and 5’UTR, intron and non-coding exon and missense variants (considered polymorphisms because highly frequent in normal population), have been excluded from further analysis. Twenty-two (11 cases and 11 controls) of the 36 patients presented at least one somatic variant, with a probable effect on protein function, in one of 26 genes analyzed by TST26, while 14 did not present any somatic mutations. A total of 38 somatic mutations (16 pathogenic, 13 likely pathogenic and nine variants of uncertain significance) were identified across 13 HCC-related genes. Coexisting somatic mutations in different genes were found in 10 of 36 patients, and six patients showed intra-tumor molecular heterogeneity (Table A1).

Most mutations were in genes involved in hepatocarcinogenesis: cell cycle regulation (8 mutations in *TP53* gene, 1 in STK11), PI3K/AKT/mTOR pathway (5 mutations in *PIK3CA* gene, 4 in *PTEN* and 3 in *KIT*) and Wnt/beta-catenin signaling pathway (8 mutations in *CTNNB1* gene,2 in *APC* gene and 1 in *CDH1*, Table A1).

There was no evidence of association of HCC recurrence with the presence of somatic mutations or with the deregulation of HCC-related pathways (data partially shown in Table 5).

Irrespective of recurrence prediction, however, our data confirmed that the presence of somatic mutations in genes associated with molecular mechanisms of hepatocarcinogenesis is associated with clinicopathological features due to damaged liver. Notably, high ALT levels were higher in the five patients presenting mutations in genes involved in the PI3K/AKT/mTOR pathway (ALT: 46.0 [39.0–186.0] vs. 34.0 [29.5–47.0], Mann–Whitney test *p*-value = 0.014) and in the eight patients presenting mutations in genes involved in Ras/MAPK pathways (ALT: 42.5 [36.5–100.5] vs. 33.0 [27.5, 46.5], *p* = 0.033); AST and ALT levels were also higher in the nine patients presenting mutations in genes involved in Wnt/beta-catenin signaling (AST: 83.0 [26.0–114.0] vs. 27.0 [23.5–34.5], *p* = 0.020; ALT: 103.0 [37.0–155.0] vs. 34.0 [31.0–40.0], *p* = 0.032), along with the need for a major resection (three patients out of nine vs. zero, Fisher’s exact test *p* = 0.012).

## 4. Discussion

Recurrence after liver resection remains a major problem that needs to be addressed [23,24,25]. In this paper, we wanted to crosscheck clinical, pathological and radiologic characteristics of patients resected for HCC with a molecular genotyping on resected hepatic parenchyma.

We searched for whether classic parameters and markers of HCC recurrence had some, if any, relationship with NGS and allelic imbalance analysis.

Firstly, we were able to depict a scenario of clinical, biochemical, radiologic and pathologic data suggestive of prognostic capacity in order to identify patients at greater risk of recurrence after liver resection. In particular, multivariate analysis confirmed total bilirubin, greater number and larger size of nodules as markers of recurrence. Those parameters seem to impact more on the risk of recurrence than the surgical technique employed to remove the tumor, as we and others previously reported [10].

Secondly, we have identified multiple molecular abnormalities in a small dataset of patients with resectable HCC. The accumulation of alterations in cancer genes and associated pathways are major causes for hepatocarcinogenesis and tumor progression.

Interestingly, a proportion of our patients had molecular aberrations associated to PI3K/AKT/mTOR pathway activation.

In our cohort, discrepancies in HCC mutation rates of major cancer genes are to be dependent on the clinical characteristics of each patient, such as stage of cancer progression, etiology of the liver disease, degree of liver dysfunction and presence/absence of an underlying chronic liver disease.

As we and others have previously reported in the analysis of the HCC recurrence after liver transplantation [19], LOH analysis even in the setting of the liver resection for HCC seem to have a potential role in the development of a therapeutic algorithm.

Looking at the NGS, the molecular analysis conducted on this small cohort of patients did not show any association between molecular markers (somatic mutations) and the recurrence of HCC after hepatectomy.

However, the data obtained agree with the current knowledge on the molecular aspects of hepatocarcinogenesis.

In particular, we analyzed four patients with HBV-related HCC, and we identified *TP53* alterations in three patients. Literature data indicate that in HBV-related HCC are frequent inactivating mutations in *TP53* and *KMT2B* genes leading to a more frequent involvement of cell cycle control apoptosis and epigenetic regulation [26].

A further analysis of four patients with alcohol-related HCC, identified LOH in *CDKN2A* and *CMM* loci (involved in overexpression of HGF) in three patients. It was shown that *TERT* promoter mutations, *CTNNB1* activating mutations, *ARID1A* inactivating mutations and alterations in *SMARCA2*, *HGF*, *RB1* and *CDKN2A* are more frequent in alcohol-related HCC [27].

In some other patients, we have found more than one somatic mutation. For example, a tumor sample with VAF of 28% for a pathogenic mutation identified in the *TP53* gene (p.Gly279Glu) coexisting with uncertain significance in *PIK3CA* (VAF of 3%) and *APC* (VAF of 5%) genes. Different cellular subclones would occur during tumor growth due to carcinogenic exposure, selective pressure from the microenvironment or the random acquisition of novel mutations. Whole exome sequencing (WES) studies have revealed that the mean number of somatic mutations in coding sequence range from 40 to 80 per tumor in HCC [28] occurs in driver and passenger genes and is not uniformly distributed through the whole tumor mass.

Alterations in driver genes contribute to tumor evolution at any stage, from cancer initiation to metastasis, while alterations in passenger have no functional consequences and occur randomly in the genome. Somatic molecular alterations in cancer are not uniformly distributed through the whole tumor mass.

The goal of clinical research in HCC management is to ameliorate the actual panel of prognostic data we have to tailor the best indication for patients with HCC candidate to liver resection.

In this respect, this study contributes to reinforce the concept that multiple alterations of cancers genes are associated with HCC progression. In particular, the evidence of a specific AI in 20 patients (six cases and 14 controls) seemed to have a protective effect on the risk of HCC recurrence. Obviously, the pre-operative knowledge of these specific or similar aspects could influence the decision-making management of HCC.

## Figures and Tables

**Table 1:** Baseline characteristics and subsequent follow-up of 124 patients who underwent hepatocellular carcinoma (HCC) surgical resection.

Total Number of Patients	124 ^§^
Age, years	69.0 (61.8–73.0)
Male sex	96 (77)
Body mass index, Kg/m^2^	25.8 (23.4–29.2)
Diabetes mellitus	39 (31)
Liver disease etiology	
Hepatitis C virus	72 (58)
Hepatitis B virus	12 (10)
Non-alcoholic steatohepatitis	18 (15)
Alcohol	9 (7)
Other/cryptogenic	4 (3)
HCC on healthy liver	9 (7)
Previous antiviral treatments (only HCV patients *N* = 72)	
None	5 (7) ^§§^
Interferon–Ribavin (IFN)	15 (21)
Direct-acting antivirals (DAA)	30 (42)
Both IFN and DAA	22 (31)
Sustained virologic response	62 (86) ^§§^
Liver cirrhosis	93 (75)
Portal hypertension	31 (25)
History of esophageal varices	
F0	99 (80)
F1	19 (15)
F2	6 (5)
Model for end-stage liver disease (MELD)	7.0 (7.0–8.0)
Child–Pugh Score	
A5	102 (82)
A6	21 (17)
B7	1 (1)
Serum bilirubin (mg/dL)	0.6 (0.4–0.9)
Serum albumin (g/dL)	3.9 (3.6–4.1)
INR median [IQR]	1.1 (1.0–1.1)
Serum creatinine (mg/dL)	0.9 (0.7–1.1)
Aspartate aminotransferase (IU/L)	30.0 (23.0–50.5)
Alanine aminotransferase (IU/L)	40.5 (29.8–67.2)
Neutrophil to lymphocyte ratio	2.0 (1.5–5.6)
Alpha-fetoprotein (ng/dL)	5.9 (3.4–20.0)
Videolaparoscopic approach	58 (47)
Major resection	14 (11)
Anatomic resection	41 (33)
Type of resection	
Right hepatectomy	11 (9)
Left hepatectomy	3 (2)
Bisegmentectomy	6 (5)
Segmentectomy	21 (17)
Wedge resection of single nodule	76 (61)
Wedge resection of multiple nodules	7 (6)
Histological grading G3–G4	39 (31)
Microvascular invasion	49 (40)
Macrovascular invasion	6 (5)
Number of HCC nodules	
1	100 (81)
2	16 (13)
3 or more	8 (6)
Size of the greater lesion	3.2 (2.0–5.4)
Tumor stage	
T1	54 (44)
T2	46 (37)
T3	22 (18)
T4	2 (2)
Cumulative number of HCC recurrence	
After 12 months	26 (21)
After 24 months	32 (26)
Probability of HCC recurrence, Kaplan–Meier estimate [95% C.I.]	
After 12 months	37% (24–47)
After 24 months	51% (35–62)

^§^ Unless otherwise stated, variables are descripted by no. (%) if categorical and by median [IQR] if numeric; ^§§^ percentages related to antiviral treatments are relative to the total number of patients with HCV etiology (*N* = 72).

**Table 2:** Univariable Cox models of time to HCC recurrence for clinical and pathologic characteristics of 124 patients (complete list is available in Table S3).

Variable	HR	95% Confidence Interval	*p*-Value
Model for end-stage liver disease	1.43	1.16–1.75	<0.001
Serum bilirubin	4.20	1.74–10.16	0.001
International normalized ratio	177.6	5.4–5872.0	0.004
Number of nodules	1.81	1.22–2.67	0.003
Size of the larger nodule	1.09	1.02–1.16	0.011
Tumor stage: T3–T4	3.05	1.45–6.42	0.003

**Table 3:** Multivariable Cox models for time to HCC recurrence in 124 patients.

Variable	HR	95% Confidence Interval	*p*-Value
HCV infection	1.96	0.91–4.24	0.085
Serum bilirubin	5.32	2.07–13.69	0.001
Number of nodules	1.63	1.12–2.38	0.011
Size of the larger nodule	1.11	1.03–1.18	0.004

**Table 4:** Univariate Cox models of time-to-recurrence for the presence of LOH in a panel of HCC-related signaling pathway.

**Gene**	**Number of** **Patients with LOH (%)**	**HR**	**95% C.I.**	***p*-Value**
*PTEN*	20 (51)	0.35	0.13–0.93	0.036
*SMAD4*	4 (10)	1.12	0.31–4.04	0.861
*CMM*	20 (51)	0.85	0.34–2.11	0.726
*CDKN2A*	15 (38)	0.57	0.21–1.51	0.256
*TP53*	30 (77)	2.65	0.76–9.22	0.124
*OGG1*	19 (49)	1.79	0.72–4.46	0.212
*L-MYC*	21 (54)	1.47	0.59–3.67	0.405
*PTCH*	14 (36)	1.31	0.50–3.41	0.581
*MCC*	11 (28)	0.46	0.16–1.27	0.134
HCC-Related Signaling Pathway				
PI3K/AKT/mTOR	20 (51)	0.35	0.13–0.93	0.036
TGF-beta	4 (10)	1.12	0.31–4.04	0.861
MAPK	20 (51)	0.85	0.34–2.11	0.726
Cell cycle regulation	37 (95)	0.51	0.07–3.94	0.520
Wnt/beta-catenin	28 (72)	0.77	0.29–2.06	0.606

**Table 5:** Frequency distribution HCC-related signaling pathway over 36 HCC patients who underwent radical liver resection.

HCC-Related Signaling Pathway	Overall (*N* = 36)	Cases (*N* = 17)	Controls (*N* = 19)	*p*-Value
PI3K/AKT/mTOR	9(25)	3(18)	6(32)	0.451
TGF-beta	2(6)	1(6)	1(5)	1.000
Wnt/beta-catenin	9(25)	5(29)	4(21)	0.706
MAPK	8(22)	3(18)	5(26)	0.695
Cell cycle regulation	9(25)	4(24)	5(26)	1.000
Inflammatory Response	1(3)	0(0)	1(5)	1.000
NOTCH1	1(3)	1(6)	0(0)	0.472

## Data Availability

The data presented in this study are available on request from the corresponding author. The data are not publicly available due to privacy policy of our institution.

## Supplementary Materials

The following are available online at https://www.mdpi.com/2072-6694/13/3/518/s1, Table S1: Microsatellites loci checked for the assessment of LOH, Table S2: Regions covered by Illumina TruSight Tumor 26, Table S3: Univariable Cox models of time to HCC recurrence for clinical and pathological characteristics of 124 patients.

## Appendix A. NGS Analysis Details

**Table A1:** Details of 38 single-nucleotide mutations detected by NGS analysis on 36 HCC patients who underwent liver resection (each row represents a detected mutation).

Gene (RefSeq) HGVSp	Patient	ACMG Classification	Variant Read Frequency	HCC-Related Signaling Pathway
***PIK3CA*** (NM_006218.3)				
p.(Glu81Lys)	Control-7	Likely Pathogenic	3%	PI3K/AKT/mTOR
p.(Leu429PhefsTer8)	Case-11	Likely Pathogenic	3%	
p.(Leu443Ser)	Control-8	Likely Pathogenic	6%	
p.(Leu49Ile)	Case-9	Likely Pathogenic	15%	
p.(Met983Ile)	Control-3	Uncertain Significance	3%	
***PTEN*** (NM_000314.6)				
c.254-1G>T	Case-11	Pathogenic	5%	PI3K/AKT/mTOR
p.(Arg173His)	Case-3	Pathogenic	4%	
p.(Tyr88Asn)	Case-11	Likely Pathogenic	19%	
p.(Tyr88Asn)	Control-9	Likely Pathogenic	39%	
***KIT*** (NM_000222.2)				
p.(Arg830Ter)	Control-8	Pathogenic	9%	PI3K/AKT/mTOR
p.(Gln556Lys)	Control-4	Likely Pathogenic	5%	
p.(His790Tyr)	Control-2	Likely Pathogenic	4%	
***MET*** (NM_001127500.2)				
p.(Trp1267Ter)	Control-1	Pathogenic	6%	MAPK
p.(Glu1145Lys)	Case-8	Uncertain Significance	3%	
***NRAS*** (NM_002524.4)				
p.(Gly180Ser)	Control-02	Uncertain Significance	4%	MAPK
***KRAS*** (NM_033360.3)				
p.(Leu79Phe)	Case-8	Uncertain Significance	4%	TGF-beta
***TP53*** (NM_000546.5)				
p.(Arg175His)	Case-7	Pathogenic	44%	Cell cycle regulation
p.(Gln375SerfsTer7)	Control-5	Pathogenic	3%	
p.(Gln375SerfsTer7)	Case-3	Pathogenic	3%	
p.(Glu294SerfsTer51)	Control-6	Pathogenic	33%	
p.(Gly279Glu)	Control-3	Pathogenic	28%	
p.(Leu194Arg)	Control-4	Pathogenic	36%	
p.(Leu206Met)	Case-2	Likely Pathogenic	4%	
p.(Gln5Arg)	Control-10	Uncertain Significance	44%	
***STK11*** (NM_000455.4)				
p.(Gly268Arg)	Case-1	Likely Pathogenic	17%	Cell cycle regulation
***CTNNB1*** (NM_001904.3)				
p.(Asp32Ala)	Case-4	Pathogenic	30%	Wnt/beta-catenin
p.(Asp32Gly)	Case-10	Pathogenic	32%	
p.(Gly34Arg)	Case-05	Pathogenic	19%	
p.(Ser33Phe)	Control-10	Pathogenic	9%	
p.(Ser45Tyr)	Control-10	Pathogenic	22%	
p.(Asp6Val)	Case-11	Likely Pathogenic	5%	
p.(Glu9Gly)	Case-11	Likely Pathogenic	9%	
p.(Ile35Ser)	Control-11	Likely Pathogenic	9%	
***APC*** (NM_000038.5)				
p.(Ala888Thr)	Case-01	Uncertain Significance	11%	Wnt/beta-catenin
p.(Gly1416Asp)	Control-03	Uncertain Significance	5%	
***CDH1*** (NM_004360.4)				
c.1716T>C(p.(Ser572=))	Control-05	Uncertain Significance	49%	Wnt/beta-catenin
***GNAS*** (NM_080425.3)				
p.(Arg844His)	Control-8	Pathogenic	14%	Inflammatory Response
***FBXW7*** (NM_018315.4)				
p.(Gln544Arg)	Case-06	Uncertain Significance	4%	NOTCH1

## Appendix B. Allelic Imbalance Analysis

**Table A2:** LOH status-according to HCC-related signaling pathway.

HCC-Related Signaling Pathway	Gene	Cases (*N* = 19)	Control (*N* = 20)	*p*-Value
PI3K/AKT/mTOR	*PTEN*			0.066
	No LOH	10 (53)	4 (20)	
	LOH	6 (32)	14 (70)	
	Non-informative	3 (16)	2 (10)	
TGF-beta	*SMAD4*			0.211
	No LOH	10 (53)	7 (35)	
	LOH	3 (16)	1 (5)	
	Non-informative	6 (32)	12 (60)	
MAPK	*CMM*			0.767
	No LOH	4 (21)	5 (25)	
	LOH	9 (47)	11 (55)	
	Non-informative	6 (32)	4 (20)	
Cell cycle regulation	*CDKN2A*			0.395
	No LOH	11 (58)	11 (55)	
	LOH	6 (32)	9 (45)	
	Non-informative	2 (11)	0 (0)	
Cell cycle regulation	*TP53*			0.451
	No LOH	3 (16)	6 (30)	
	LOH	16 (84)	14 (70)	
	Non-informative	0(0)	(0)	
Cell cycle regulation	*OGG1*			0.223
	No LOH	6 (32)	5 (25)	
	LOH	11 (58)	8 (40)	
	Non-informative	2 (11)	7 (35)	
Wnt/beta-catenin	*L-MYC*			0.751
	No LOH	8 (42)	10 (50)	
	LOH	11 (58)	10 (50)	
	Non-informative	2 (11)	7 (35)	
Wnt/beta-catenin	*PTCH*			1.000
	No LOH	10 (53)	10 (50)	
	LOH	7 (37)	7 (35)	
	Non-informative	2 (11)	3 (15)	
Wnt/beta-catenin	*MCC*			0.776
	No LOH	10 (53)	8 (40)	
	LOH	5 (26)	6 (30)	
	Non-informative	4 (21)	6 (30)	

## References

[1] Bray F., Ferlay J., Soerjomataram I., Siegel R.L., Torre L.A., Jemal A. (2018). Global Cancer Statistics 2018: GLOBOCAN Estimates of Incidence and Mortality Worldwide for 36 Cancers in 185 Countries. CA Cancer J. Clin..

[2] Galle P.R., Forner A., Llovet J.M., Mazzaferro V., Piscaglia F., Raoul J.-L., Schirmacher P., Vilgrain V. (2018). EASL Clinical Practice Guidelines: Management of Hepatocellular Carcinoma. J. Hepatol..

[3] Gruttadauria S. (2020). Minimally Invasive Liver Surgery in the Setting of the Hepatocellular Carcinoma. J. Laparoendosc. Adv. Surg. Tech. Videoscop..

[4] Gruttadauria S., Vasta F., Minervini M.I., Piazza T., Arcadipane A., Marcos A., Gridelli B. (2005). Significance of the Effective Remnant Liver Volume in Major Hepatectomies. Am. Surg..

[5] Tropea A., Barbara M., Calamia S., Lomaglio L., Bonsignore P., di Francesco F., Pagano D., Gruttadauria S. (2020). Laparoscopic Microwave Thermal Ablation for the Treatment of Hepatocellular Carcinoma in Chronic Hepatic Patients. J. Laparoendosc. Adv. Surg. Tech. Videoscop..

[6] Pagano D., Ricotta C., Barbàra M., Cintorino D., di Francesco F., Tropea A., Calamia S., Lomaglio L., Terzo D., Gruttadauria S. (2020). ERAS Protocol for Perioperative Care of Patients Treated with Laparoscopic Nonanatomic Liver Resection for Hepatocellular Carcinoma: The ISMETT Experience. J. Laparoendosc. Adv. Surg. Tech. Videoscop..

[7] Mazzaferro V., Citterio D., Bhoori S., Bongini M., Miceli R., de Carlis L., Colledan M., Salizzoni M., Romagnoli R., Antonelli B. (2020). Liver Transplantation in Hepatocellular Carcinoma after Tumour Downstaging (XXL): A Randomised, Controlled, Phase 2b/3 Trial. Lancet Oncol..

[8] Xu X.-F., Xing H., Han J., Li Z.-L., Lau W.-Y., Zhou Y.-H., Gu W.-M., Wang H., Chen T.-H., Zeng Y.-Y. (2019). Risk Factors, Patterns, and Outcomes of Late Recurrence After Liver Resection for Hepatocellular Carcinoma: A Multicenter Study From China. JAMA Surg..

[9] Chan A.W.H., Zhong J., Berhane S., Toyoda H., Cucchetti A., Shi K., Tada T., Chong C.C.N., Xiang B.-D., Li L.-Q. (2018). Development of Pre and Post-Operative Models to Predict Early Recurrence of Hepatocellular Carcinoma after Surgical Resection. J. Hepatol..

[10] Gruttadauria S., Pagano D., Corsini L.R., Cintorino D., Petri S.L., Calamia S., Seidita A., di Francesco F. (2020). Impact of Margin Status on Long-Term Results of Liver Resection for Hepatocellular Carcinoma: Single-Center Time-to-Recurrence Analysis. Updates Surg..

[11] Ding X., He M., Chan A.W.H., Song Q.X., Sze S.C., Chen H., Man M.K.H., Man K., Chan S.L., Lai P.B.S. (2019). Genomic and Epigenomic Features of Primary and Recurrent Hepatocellular Carcinomas. Gastroenterology.

[12] Rossi J.Z., Villanueva A., Nault J.-C., Llovet J.M. (2015). Genetic Landscape and Biomarkers of Hepatocellular Carcinoma. Gastroenterology.

[13] Llovet J.M., Rossi J.Z., Pikarsky E., Sangro B., Schwartz M., Sherman M., Gores G. (2016). Hepatocellular Carcinoma. Nat. Rev. Dis. Primers.

[14] Niu Z.-S., Niu X.-J., Wang W.-H. (2016). Genetic Alterations in Hepatocellular Carcinoma: An Update. World J. Gastroenterol..

[15] Schulze K., Nault J.-C., Villanueva A. (2016). Genetic Profiling of Hepatocellular Carcinoma Using Next-Generation Sequencing. J. Hepatol..

[16] Bielski C.M., Donoghue M.T.A., Gadiya M., Hanrahan A.J., Won H.H., Chang M.T., Jonsson P., Penson A.V., Gorelick A., Harris C. (2018). Widespread Selection for Oncogenic Mutant Allele Imbalance in Cancer. Cancer Cell.

[17] Thorgeirsson S.S., Grisham J.W. (2002). Molecular Pathogenesis of Human Hepatocellular Carcinoma. Nat. Genet..

[18] Marsh J. (2003). Genotyping of Hepatocellular Carcinoma in Liver Transplant Recipients Adds Predictive Power for Determining Recurrence-Free Survival. Liver Transplant..

[19] Pagano D., Barbera F., Conaldi P.G., Seidita A., di Francesco F., di Carlo D., Barbàra M., Tuzzolino F., Luca A., Gruttadauria S. (2019). Role of Allelic Imbalance in Predicting Hepatocellular Carcinoma (HCC) Recurrence Risk After Liver Transplant. Ann. Transplant..

[20] Dong L., Peng L., Ma L., Liu D., Zhang S., Luo S., Rao J., Zhu H., Yang S., Xi S. (2020). Heterogeneous Immunogenomic Features and Distinct Escape Mechanisms in Multifocal Hepatocellular Carcinoma. J. Hepatol..

[21] Wing M.R., Reeser J.W., Smith A.M., Reeder M., Martin D., Jewell B.M., Datta J., Miya J., Monk J.P., Mortazavi A. (2017). Analytic Validation and Real-Time Clinical Application of an Amplicon-Based Targeted Gene Panel for Advanced Cancer. Oncotarget.

[22] Giardina T., Robinson C., Iacopetta F.G., Millward M., Iacopetta B., Spagnolo D., Amanuel B. (2018). Implementation of next Generation Sequencing Technology for Somatic Mutation Detection in Routine Laboratory Practice. Pathology.

[23] Maruzzelli L., Miraglia R., Caruso S., Milazzo M., Mamone G., Gruttadauria S., Spada M., Luca A., Gridelli B. (2010). Percutaneous Endovascular Treatment of Hepatic Artery Stenosis in Adult and Pediatric Patients After Liver Transplantation. Cardiovasc. Interv. Radiol..

[24] Gruttadauria S., Chaumet M.S.G., Pagano D., Marsh J.W., Bartoccelli C., Cintorino D., Arcadipane A., Vizzini G., Spada M., Gridelli B. (2011). Impact of Blood Transfusion on Early Outcome of Liver Resection for Colorectal Hepatic Metastases: Blood Transfusion on Liver Resection. J. Surg. Oncol..

[25] Marrone G. (2012). Multidisciplinary Imaging of Liver Hydatidosis. World J. Gastroenterol..

[26] Schulze K., Imbeaud S., Letouzé E., Alexandrov L.B., Calderaro J., Rebouissou S., Couchy G., Meiller C., Shinde J., Soysouvanh F. (2015). Exome Sequencing of Hepatocellular Carcinomas Identifies New Mutational Signatures and Potential Therapeutic Targets. Nat. Genet..

[27] Totoki Y., Tatsuno K., Covington K.R., Ueda H., Creighton C.J., Kato M., Tsuji S., Donehower L.A., Slagle B.L., Nakamura H. (2014). Trans-Ancestry Mutational Landscape of Hepatocellular Carcinoma Genomes. Nat. Genet..

[28] Ally A., Balasundaram M., Carlsen R., Chuah E., Clarke A., Dhalla N., Holt R.A., Jones S.J.M., Lee D., Ma Y. (2017). Comprehensive and Integrative Genomic Characterization of Hepatocellular Carcinoma. Cell.

