# Supplementary material for: Clinical and Molecular-Based Approach in the Evaluation of Hepatocellular Carcinoma Recurrence after Radical Liver Resection
Source: Cancers (Basel). 2021 Jan 29;13(3):518. doi: 10.3390/cancers13030518 (PMC7866287; doi:10.3390/cancers13030518)
Supplement: Supplementary file 1 [file cancers-13-00518-s001.pdf]

cancers

MDPI

Article

Clinical and Molecular-Based Approach in the Evaluation of the Risk of Hepatocellular Carcinoma Recurrence After Radical Liver Resection

Salvatore Gruttadauria, Floriana Barbera, Pier Giulio Conaldi, Duilio Pagano, Rosa Liotta, Enrico Gringeri , Roberto Miraglia, Gaetano Burgio, Marco Barbara, Giada Pietrosi, Calogero Cammà and Fabrizio Di Francesco

Supplementary Materials:

Table S1. Microsatellites loci checked for the assessment of LOH.

Gene	Locus	Microsatellite	HCC-related Signaling Pathway
L-MYC	1p33	D1S162	Wnt/beta-catenin
L-MYC	1p34	MYCL5NT	
L-MYC	1p35	D1S1161	
CMM	1p36	D1S407	MAPK
OGG1	3p24	D3S2303	Cell cycle regulation
MCC	5p21	D5S592	Wnt/beta-catenin
CDKN2A	9p21	D9S251	Cell cycle regulation
CDKN2A	9p21	D9S254	
PTCH	9q22	D9S252	Wnt/beta-catenin
PTEN	10q23	D10S520	PI3K/AKT/mTOR
PTEN	10q23	D10S1173	
TP53	17p13	D17S974	Cell cycle regulation
TP53	17p13	TP53-L1	
TP53	17p13	D17S1289	
TP53	17p23	D17S786	
TP53	17p23	D17S516	
SMAD4	18q21	D18S814	TGF-beta

Table S2. Regions covered by Illumina TruSight Tumor 26.

Gene Symbol	Accession Number	Exons Covered	Number of Amplicons to Exon Coverage
AKT1	NG_012188.1	2	1
ALK	NG_009445.1	23	1
APC	NG_008481.4	15	14
BRAF	NG_007873.3	11,15	3
CDH1	NG_008021.1	8,9,12	6
CTNNB1	NG_013302.2	2	2
EGFR	NG_007726.3	18,19,20,21	7
ERBB2	NG_007503.1	20	2

FBXW7	NG_029466.2	7,8,9,10,11	13
FGFR2	NG_012449.2	6	2
FOXL2	NG_012454.1	1	1
GNAQ	NG_027904.2	4,5,6	6
GNAS	NG_016194.2	6,8	2
KIT	NG_007456.1	9,11,13,17,18	9
KRAS	NG_007524.1	1,2,3,4	8
MAP2K1	NG_008305.1	2	1
MET	NG_008996.1	1,4,13,15,16,17,18,20	22
MSH6	NG_007111.1	5	3
NRAS	NG_007572.1	1,2,3,4	8
PDGFRA	NG_009250.1	11,13,17	5
PIK3CA	NG_012113.2	1,2,7,9,20	15
PTEN	NG_007466.2	1,2,3,4,5,6,7,9	17
SMAD4	NG_013013.2	8,11	5
SRC	NG_023033.1	10	2
STK11	NG_007460.2	1,4,6,8	7
TP53	NG_017013.2	2,3,4,5,6,7,8,9,10,11	16

Table S3. Univariable Cox models of time to HCC recurrence for clinical and pathological characteristics of 124 patients.

**Variable**	**HR**	**Lower95**	**Upper95**	***p*-value**
Age	0.97	0.94	1.00	0.088
Sex: M	1.44	0.60	3.45	0.416
BMI	1.03	0.96	1.10	0.361
Diabetes: 1	0.72	0.34	1.52	0.387
Etiology: HBV	0.33	0.08	1.41	0.135
Etiology: NASH	0.64	0.22	1.85	0.409
Etiology: Alcohol	1.15	0.27	4.95	0.85
Etiology: Healthy	2.43	0.91	6.43	0.075
HCV: 1	1.24	0.63	2.43	0.53
HBV: 1	0.33	0.08	1.37	0.126
ETOH: 1	0.94	0.29	3.09	0.922
NASH: 1	0.71	0.28	1.83	0.483
Cirrhosis: 1	0.79	0.36	1.73	0.555
Portal hypertension: 1	0.96	0.47	1.94	0.902
Esophageal varices: 1	1.05	0.52	2.12	0.901
MELD	1.43	1.16	1.75	<0.001
Child-Pugh Score: 6–7	0.97	0.42	2.20	0.934
Serum Bilirubin	4.20	1.74	10.16	0.001
Serum Albumin	1.49	0.74	3.00	0.266
International Normalized Ratio	177.57	5.37	5872.00	0.004
INR > 1.1	2.68	1.34	5.36	0.005
Serum Creatinine	0.63	0.23	1.77	0.384
Aspartate aminotransferase	1.00	1.00	1.00	0.125
Alanine aminotransferase	1.00	0.99	1.01	0.682
Platelets	1.00	0.99	1.00	0.642
Neutrophils	1.08	0.89	1.31	0.459
Lymphocytes	1.26	0.92	1.72	0.146
Neutrophil to lymphocyte ratio (NLR)	0.92	0.71	1.20	0.551
Hemoglobin	1.01	0.84	1.21	0.934
Alpha-fetoprotein	1.00	1.00	1.00	0.762
Video laparoscopic resection: LPS	1.00	0.53	1.91	0.99

**Variable**	**HR**	**Lower95**	**Upper95**	***p*-value**
Major resection	2.15	0.97	4.74	0.058
Number of involved segments	1.49	1.15	1.95	0.003
Anatomic resection	1.58	0.83	3.01	0.167
Grading: G3-G4	1.15	0.59	2.24	0.673
Microvascular invasion: SI	1.90	0.99	3.61	0.052
Macrovascular invasion: SI	1.01	0.31	3.31	0.985
Number of nodules	1.81	1.22	2.67	0.003
Diameter	1.09	1.02	1.16	0.011
pT: T3-T4	3.05	1.45	6.42	0.003
